# Potential anti-inflammatory role of 2-chloroadenosine treatment during acute lung inflammation in BALB/c mice suffering from *Klebsiella pneumoniae *B5055-induced acute lung infection

**DOI:** 10.1186/cc12967

**Published:** 2013-11-05

**Authors:** Vijay Kumar, Kusum Harjai, Sanjay Chhibber

**Affiliations:** 1 Department of Microbiology, Panjab University, Chandigarh, India

## Background

Acute lung inflammation (ALI) is a life-threatening pathology and can develop during the course of several clinical conditions such as pneumonia, acid aspiration or sepsis. Adenosine plays a significant role in controlling acute inflammation via binding to A2A receptors on inflammatory cells; that is, neutrophils or macrophages. The present study was designed to evaluate the anti-inflammatory and immunomodulatory effects of 2-chloroadenosine (2-CADO), alone or in combination with amoxicillin/clavulanic acid (AMC), in *Klebsiella pneumoniae *B5055-induced acute lung infection in mice.

## Materials and methods

Acute lung infection in mice was induced by directly instilling the selected dose (10^4 ^colony-forming units/ml) of bacteria intranasally. Histopathological examination of the lungs was performed to reveal neutrophil infiltration into the lung alveoli. In addition to the major proinflammatory cytokines TNFα and IL-1α, levels of the anti-inflammatory cytokine IL-10 were also determined by ELISA.

## Results

Intranasal instillation of bacteria caused profound neutrophil infiltration into the lung alveoli as well as a significant increase in the levels of proinflammatory mediators (that is, TNFα and IL-1α). However, intravenous administration of 2-CADO 10 μg/kg/day, alone or in combination with an antibiotic (that is, AMC 20 μg/ml/day i.p. 1 day after establishment of infection), significantly decreased neutrophil infiltration into the lung alveoli. A significant decrease in TNFα and IL-1α along with elevation of IL-10 levels in the lung homogenate of mice with acute lung infection was observed upon treatment with 2-CADO alone, with no significant decrease in bacterial counts. Moreover in combination with AMC, 2-CADO exhibited its immunomodulatory action in acute lung infection and prevented ALI observed during acute bacterial pulmonary infection, whilst an antibacterial action was exhibited by AMC.

## Conclusions

2-CADO proved a potent immunomodulatory agent during acute Gram-negative bacteria-induced ALI and exhibited its anti-inflammatory and immunomodulatory potential even in the presence of antibiotics. Thus, it has a potential to be used as an adjunct immunomodulatory agent during acute inflammatory conditions like ALI or sepsis.

